# How to Leave Church

**Published:** 1855-06

**Authors:** 


### HOW TO LEAVE CHURCH,

Shut Your Mouth and Move on.—That tells the whole story. To spread out all the advantages, as the subject merits, would require several pages. City human nature is to be always in a hurry—it is a necessity. We must be in a hurry, or out of bread. Five minutes is half a dollar or half a thousand to some business man in New York, in character or money in almost any day in the year. Banks are closed at the moment. All the great lines of rail cars and steamboats leave at the moment, and that moment lost, is twelve or twenty-four hours gone forever. Many a reader will acknowledge a decided feeling of irritation or impatience, if not actual mental anathema, at the inconsiderate practice of many church goers, of stopping to shake hands in the aisles, at the close of religious services. This leads to exchange of compliments, and inquiries and answers, standing still the while, and thus hindering all the crowd behind them. Others reserve their loitering until they reach the door-steps, and then take a deliberate view of the throng before them, apparently satisfied with having reached the fresh air themselves. There is scarcely ever a religious assembly, at which there is not one or more persons whom some urgent business—some sick child, or suffering parent does not call away in all haste, compatible with the decencies of the occasion, and no one has a right to deprive me of the earliest return to loved ones at home. Not only is that minute lost to me,—and how long is even a minute to the suffering expectant one there—but an equal time is lost to the fifty or five hundred who may be behind me. To those who aim to "*Do justly and love mercy*," I commend reflection on this point.

Besides, when I have heard a good discourse,—when I have been really fed in the sanctuary—I don't want to be irritated out of it, by a thoughtless loiterer, who thus makes me run the risk of losing an engagement or missing an appointment. Then again, when one has been warmed up religiously by a heart-searching gospel sermon, and his whole soul is subdued by the soothing influence of Bible preaching, it falls harshly indeed upon the ear, to have remarks made, whether of idle compliment, or cold formality, or profane mirth. Not long since, in a Fifth Avenue church, I was obliged to listen to a lady in the aisle, remarking to a gentleman, on the comparative merits of a dinner of soup or one of mush and milk. She averred they were both excellent—for poor people; for I soon learned she was connected with a benevolent soup society, or a soup benevolent society. That did alter the case some; for it is an old time maxim of mine, that *the case being altered, alters the case.* Still, altered as it was, the sense of the ridiculous had got such an ascendancy, that every idea of the sermon, if it had any, took to itself wings and flew away, and what is more, they never came back again; and for hours after, there were floating about in my brain, images of poverty and Fifth Avenue, gospel soup, mush preaching, philanthropic barege, muslin de'laines, cashmere shawls. The preaching of that day was lost to me. I had understood, before, that the gospel was "*bread*"—that it was the "*pure milk of the word;*" and on one occasion, in the little English chapel in Paris, not far from the Champ D'Elysees, that it was the real *Eau De vie*, and ought to be drank freely; but that it should be mixed up in my mind with such things as "soup," "stirabout," "mush," yes, vulgar "*mush*," is too bad.

But near that lady there may have been another, upon whose heart the sermon had fallen with penetrating power, whom it had *almost persuaded to be a Christian;* and as he was slowly passing out, he might have been just on the point of deciding to *be a Christian now.* Would not the sound have fallen upon his ears as Milton's doors, turning upon their rusty hinges, "grating harsh thunder." The sound of MUSH!

Not very many years ago, a young man had been deeply impressed with the importance of religion, and concluded that after the service, he would call upon the minister for conversation and instruction; but a person near him was overheard to say, "What a tedious sermon that was;" he immediately reflected that surely his feelings were overwrought, and that he had attached more importance to the discourse than was merited. The result was, he did not call upon the minister, and died several years afterwards, never having had a return of those serious feelings. Humanly speaking, this man was almost saved; died, having been in sight of heaven—but never reached there. He died not far from home; but never got nearer!

But this subject has a bearing on health, of greater importance than many might imagine. If churches are chilly, the sooner you get out after service, and walk briskly, so as to wake up the circulation, the greater will be your chances of not taking cold.

Usually in cold weather, churches become warm—almost oppressively so—towards the close of the services; the thermometer approaching seventy degrees, causing in many actual perspiration. If you go immediately into the street, and the sun gives no sign of thaw, there is a change in a moment's time, of some forty degrees. Under such circumstances, walking slow and conversing, the raw wind penetrates the clothing and chills the skin, while a cold dash of air is thrown in upon the tender lungs at each word or two or sentence, through the open mouth; thus in a moment's time checking external perspiration and chilling the whole lungs. But suppose you walk fast; that creates a vigorous throwing of the warm blood to the skin, to the surface, and counteracts the effects of a cold wind, to a very considerable extent; while, if the mouth be closed, the cold air is not at once thrown in upon the lungs, enervated by breathing hot air for two hours; but it passes up the nostrils, and making a circuit through the head, down to the throat, is thus thoroughly warmed before it gets to the lungs, and causes no shock at all. It is the neglect of this simple precaution, which originates colds, and not unfrequently fatal ones. Many persons are kept from going to church because they "*are sure to take cold there;*" though I have not known a person to avoid the theatre, the concert, or the opera on that account.

I do not advise by any means that persons should bolt out of church as if the house were on fire. For common decorum requires a pause after the benediction has been fully pronounced; but when you have once left your pew, move on with decent pace; make no pauses: engage in no conversation with any one, until you have reached the side walk, and if when you get there, your sense of propriety allows you to stand still, obstructing and staring at passers by, be it so. It is not quite so objectionable as barricading a church aisle.

Taking every view of the case, a short practice will convince any observer of the advantages physical, polite-i-cal, and religious, of following the advice at the head of this article—"*In leaving church, shut your mouth and move on.*"

